# End-of-Life Care Stress, Attitudes Toward End-of-Life Care, and End-of-Life Care Performance as Predictors of Job Satisfaction Among Nurses Working in Hospitals in South Korea

**DOI:** 10.3390/healthcare13233179

**Published:** 2025-12-04

**Authors:** Jooyoung Cheon

**Affiliations:** College of Nursing, Sungshin Women’s University, Seoul 02844, Republic of Korea; jcheon@sungshin.ac.kr

**Keywords:** nurses, terminal care, job satisfaction’ hospitals

## Abstract

**Background/Objectives**: This study explored end-of-life care stress, attitudes toward end-of-life care, and end-of-life care performance as predictors of job satisfaction among hospital nurses. **Methods**: A descriptive cross-sectional design was employed to assess job satisfaction among nurses with end-of-life care experience in tertiary and general hospitals in South Korea. A convenience sample of 215 nurses was recruited. Eligibility criteria included at least 3 months of experience as a direct care nurse and having provided care to terminally ill patients at least once. Data were collected through an online survey. The study variables included end-of-life care stress, attitudes toward end-of-life care, end-of-life care performance, and job satisfaction. Data were analyzed using descriptive statistics, ANOVA, Pearson correlation coefficients, and hierarchical regression analysis. **Results**: Attitudes toward end-of-life care (β = 0.277, *p* < 0.001) and end-of-life care performance (β = 0.339, *p* < 0.001) were significant predictors of job satisfaction, with being enrolled in a master’s nursing program (β = 0.228, *p* < 0.001) also contributing positively. End-of-life care stress showed no direct association with job satisfaction. The final model explained 29.4% of the variance in job satisfaction (adjusted R^2^ = 0.294). **Conclusions**: End-of-life care performance was the strongest predictor of job satisfaction, suggesting that nurses’ perceived competence enhances professional fulfillment. Positive attitudes toward end-of-life care further strengthen satisfaction. Continuous education and supportive organizational environments are essential to enhance nurses’ competence, attitudes toward end-of-life care, and quality of end-of-life care.

## 1. Introduction

Delivering high-quality end-of-life (EOL) care in acute hospital settings has become increasingly important as the prevalence of chronic and terminal illnesses continues to rise. Nurses play a central role in EOL care, providing not only symptom management and physical comfort but also emotional, spiritual, and family support for patients facing the end of life [1]. However, caring for dying patients in high-acuity hospital environments exposes nurses to intense psychological, moral, and organizational challenges that may compromise their well-being, job satisfaction, and the quality of care provided [2,3,4].

Nurses in tertiary and general hospitals are routinely on the frontlines of EOL care. They manage heavy workloads, make complex clinical decisions, and are frequently exposed to patient suffering and death [2,3,4]. This issue is particularly pronounced in South Korea, where in-hospital mortality rates remain among the highest globally, reaching 75.1% in 2024 [5]. Korean nurses in acute care settings, especially in tertiary hospitals, participate in every stage of critical and EOL care. However, their work is constrained by strong family involvement in clinical decisions, hierarchical organizational cultures, limited openness in discussing death, and sustained workload pressures [6,7,8]. These constraints intensify EOL care stress, shape nurses’ attitudes toward caring for dying patients, and influence their ability to deliver effective EOL care. Ultimately, these contextual challenges may closely affect nurses’ job satisfaction and broader professional quality of life [8,9,10,11,12,13].

Established theoretical perspectives emphasize that nurses’ job outcomes result from the dynamic interplay between job demands and the resources available to manage them [14,15]. Within this framework, the experiences of nurses providing EOL care reflect these interconnected elements. Stress associated with EOL care functions as a job demand that can diminish job satisfaction. In contrast, positive attitudes toward EOL care and higher levels of competence operate as personal resources that enhance professional meaning, strengthen a sense of efficacy, and support purpose and fulfillment in their work [14,15].

Job satisfaction is a critical outcome for staff retention and effective clinical performance. It is closely linked to patient care quality, professional fulfillment, and organizational stability [12,16]. Among nurses, job satisfaction is a multifaceted construct encompassing intrinsic factors such as professional accomplishment, meaning, and relationships, in addition to extrinsic factors such as workload, leadership, and institutional support [16,17]. In emotionally demanding contexts such as EOL care, job satisfaction is particularly influenced by nurses’ perceived stress, competence, and attitudes toward death and dying [16,17,18].

High levels of EOL care stress have been associated not only with burnout but also with reduced job satisfaction and increased turnover intentions [6,10,19,20]. Nurses often cope with patient deaths by emotionally distancing themselves or suppressing their feelings, reflecting ongoing struggles to manage grief and loss [10,21]. Some nurses even consider transferring to less death-oriented departments or leaving the profession altogether [10]. Repeated exposure to patient deaths can also lead to helplessness, moral distress, or existential fatigue, further diminishing professional morale [8,10].

Attitudes toward EOL care shape how nurses provide EOL care, influencing empathy, communication, and the integration of holistic practice [18,20,22]. Positive attitudes toward EOL care have been shown to foster professional growth, enhance relationships with patients and families, and improve the emotional climate of care units [16]. Nurses who approach death with acceptance and compassion demonstrate stronger emotional regulation and greater professional efficacy, resulting in lower stress and burnout [2,23]. Conversely, negative or avoidant attitudes—often reinforced by inadequate EOL education or cultural discomfort with discussing death—can heighten moral distress and erode confidence in providing appropriate care [6,22,24].

EOL care performance—encompassing symptom management, communication, emotional support, and spiritual care—represents a core competency that integrates technical proficiency with compassionate practice [24,25,26]. High performance in these domains enables nurses to deliver dignified, patient-centered care, strengthens their sense of professional identity, and contributes to job satisfaction [16,27]. Research indicates that higher perceived competence in EOL care is associated with reduced anxiety about death and greater resilience when coping with patient loss [11,18]. Moreover, educational interventions that enhance knowledge and self-competence are linked to more positive attitudes, improved communication, and greater psychological readiness for EOL care [24,25,28]. Collectively, these findings underscore that fostering both positive attitudes and performance capacities is essential for maintaining high-quality EOL care and sustaining nurses’ professional satisfaction.

Despite growing awareness of EOL care in Korean culture, there remains a scarcity of empirical research examining how EOL-related factors, including EOL care stress, attitudes toward EOL care, and EOL care performance, jointly influence job satisfaction among hospital nurses in South Korea. Understanding these relationships is essential for designing interventions that enhance both the quality of EOL care and nurses’ professional well-being. Therefore, this study aimed to investigate the influence of EOL care stress, attitudes toward EOL care, and EOL care performance on job satisfaction among nurses working in tertiary and general hospitals. By identifying these associations, the findings will provide evidence-based insights to guide institutional and educational strategies that promote job satisfaction and strengthen dignified EOL care in acute care settings.

## 2. Materials and Methods

### 2.1. Study Design

A descriptive cross-sectional design using an online survey was employed to identify factors associated with nurses’ job satisfaction.

### 2.2. Participants and Data Collection

A convenience sample of 215 nurses working in tertiary and general hospitals in Seoul and Gyeonggi-do, South Korea, was recruited for this study. The inclusion criteria were as follows: (1) nurses who understood the purpose of the study and voluntarily agreed to participate; (2) nurses with at least 3 months of experience as direct care providers in tertiary or general hospitals in Seoul and Gyeonggi-do; and (3) nurses who had cared for terminally ill patients at least once. The exclusion criteria were as follows: (1) nurses who had not provided direct patient care for at least one shift, such as those working in operating rooms, central supply rooms, or outpatient clinics, and (2) nurses in administrative positions not involving direct nursing care.

The primary researcher initially contacted nurses from three different hospitals, two in Seoul and one in Gyeonggi-do, to ensure variation in clinical departments and work environments, and provided them with the URL to the online questionnaire via mobile communication. The survey link was shared through general ward networks and alumni groups rather than academic or professional development-focused communities, which enabled participation from nurses with diverse backgrounds and motivations.

The online survey began in the second week of June 2024 and continued through the last week of June. The questionnaire, created using Google Forms (Google LLC, Mountain View, CA, USA), was distributed via KakaoTalk (Kakao Corp., Jeju, Republic of Korea), a widely used social communication platform in Korea. The first page of the questionnaire explained the study’s purpose, contents, and voluntary participation. It explicitly stated that participants could withdraw at any time and assured them that all responses would remain anonymous and confidential. The survey proceeded only after participants provided informed consent.

The required sample size was calculated using G*Power software (version 3.1.9.7; Heinrich-Heine-Universität Düsseldorf, Düsseldorf, Germany). For a multiple regression analysis with an effect size of 0.15, a power of 0.95, a significance level of 0.05, and 16 predictors, the minimum required sample size was estimated to be 204. To account for an anticipated 20% dropout rate and incomplete responses, the target sample size was set at 250 participants. An unspecified number of potential participants were invited to complete the questionnaire via the Google Form link. Of these, 252 individuals consented and submitted responses. Among them, 24 were excluded because they were not employed at general hospitals in Seoul or Gyeonggi-do, five were excluded because they were head nurses, and eight were excluded because they worked in departments without direct patient care responsibilities. Consequently, data from 215 participants were included in the final analysis, yielding a valid response rate of 85.3%.

### 2.3. Measurements

General characteristics (sociodemographic and clinical factors) of the study participants included age, sex, marital status, religious affiliation, educational level, subjective economic status, hospital region, hospital size, clinical career, type of working unit, position, participation in education on EOL care, and participation in EOL care during clinical practice.

EOL care stress was measured using Lee’s [29] 40-item scale, which includes seven subscales: patient/family attitudes, time constraints, bereavement burden, duty overload, relational conflict, insufficient knowledge or skills, and medical limitations. Items were rated on a 5-point Likert scale (1 = strongly disagree to 5 = strongly agree), with total scores ranging from 40 to 200; higher scores indicated greater stress. Cronbach’s α was 0.93 in previous studies [26,29] and 0.94 in the present study.

Attitudes toward EOL care were assessed using the Korean version of Frommelt’s 30-item scale translated by Cho and Kim [25]. Each item was rated on a 4-point Likert scale, yielding total scores ranging from 30 to 120; higher scores indicated more positive attitudes. Cronbach’s α was 0.82–0.86 in previous studies [28,30] and 0.77 in this study.

EOL care performance was measured using a tool developed by Park and Choi [31]. The instrument consists of 22 items rated on a 4-point Likert scale, with total scores ranging from 22 to 88. Higher scores indicated better EOL care performance. The tool demonstrated strong reliability, with Cronbach’s α values of 0.85–0.93 in previous studies [26,31] and 0.91 in the current one.

Job satisfaction was assessed using the Job Satisfaction Scale for Clinical Nurses (JSS-CN) developed by Lee et al. [32]. This 33-item instrument comprises six subscales: recognition from the organization and professional achievement, personal maturation through the nursing profession, interpersonal interaction with respect and recognition, accomplishment of accountability as a nurse, display of professional competency, and stability and job worth. Each item was rated on a 5-point Likert scale, producing a total score ranging from 33 to 165, with higher scores reflecting greater job satisfaction. The tool demonstrated excellent reliability, with a Cronbach’s α of 0.95 in the original study [32]; in the current study, the total Cronbach’s α was 0.94.

### 2.4. Data Analysis

Data were analyzed using IBM SPSS Statistics version 29.0 (SPSS/WIN 29.0) (IBM Corp., Armonk, NY, USA). Descriptive statistics (mean and standard deviation) were calculated. Independent *t*-tests, one-way ANOVA, and post hoc Scheffé tests were conducted to analyze differences in job satisfaction according to nurses’ general characteristics. Pearson correlation coefficients were then computed to examine relationships among EOL care stress, attitudes toward EOL care, EOL care performance, and job satisfaction. Finally, hierarchical regression analysis was performed to identify factors associated with job satisfaction among nurses with EOL care experience.

### 2.5. Ethical Considerations

The study protocol was approved by the Institutional Review Board of the university to which the author is affiliated. The online questionnaire provided detailed information about the study, including its purpose, duration, procedures, potential risks and benefits, personal information protection measures, compensation for potential losses, and the right to withdraw consent. The first page of the online information sheet clearly described the study’s objectives and voluntary participation. To ensure confidentiality, no personally identifying information was collected. All data were securely stored on the principal investigator’s password-protected computer, accessible only to the investigator.

## 3. Results

The mean age of participants was 31.80 years (standard deviation [SD] = 6.99; median = 29.00) (Table 1). The mean length of clinical career was 7.77 years (SD = 6.61), with a median of 5.25 years. A significant difference in job satisfaction was observed by educational level (F = 11.759, *p* < 0.001). Post hoc analysis indicated that nurses enrolled in a master’s nursing program reported higher job satisfaction (122.79 ± 15.16) than nurses with a bachelor’s degree (109.38 ± 15.71). Nurses with a master’s degree (111.63 ± 14.13) did not differ significantly from either group. No clinical factor showed a statistically significant association with job satisfaction.

As shown in Table 2, participants reported a mean EOL care stress score of 154.94 (SD = 21.07) on a scale ranging from 40 to 200. The mean score for attitudes toward EOL care was 89.56 (SD = 7.23) out of a possible 30 to 120. EOL care performance averaged 55.72 (SD = 9.98), with a potential range of 22 to 88. The mean score of job satisfaction was 111.90 (SD = 16.33) on a scale of 33 to 165.

Correlation analyses were performed to examine the relationships among the study variables (Table 3). Age showed weak positive correlations with both EOL care stress (r = 0.213, *p* = 0.002) and job satisfaction (r = 0.161, *p* = 0.018). EOL care stress demonstrated a weak but significant positive correlation with EOL care performance (r = 0.151, *p* = 0.027), and it was not significantly related to attitudes toward EOL care (r = −0.104, *p* = 0.128) or job satisfaction (r = 0.018, *p* = 0.798). Attitudes toward EOL care showed moderate positive correlations with job satisfaction (r = 0.354, *p* < 0.001) and with EOL care performance (r = 0.264, *p* < 0.001). Similarly, EOL care performance demonstrated a moderate positive correlation with job satisfaction (r = 0.408, *p* < 0.001).

Hierarchical regression analysis was conducted to identify factors associated with job satisfaction (Table 4). Prior to analysis, the assumptions of normality, homoscedasticity, independence of residuals, and multicollinearity were verified. The Durbin–Watson value was 1.557, indicating no autocorrelation of residuals. Tolerance values ranged from 0.161 to 0.959, and the variance inflation factor (VIF) ranged from 1.043 to 6.207, all within acceptable thresholds (tolerance ≥ 0.1; VIF < 10), confirming the absence of multicollinearity. Residual plots supported the assumptions of normality and equal variance. In Model 1, which included sociodemographic variables, being enrolled in a master’s nursing program (vs. bachelor’s degree) was significantly associated with higher job satisfaction (B = 12.876, β = 0.302, *p* < 0.001), and male sex also showed a positive association (B = 10.594, β = 0.143, *p* = 0.033). This model explained 10.7% of the variance (R^2^ = 0.141, adjusted R^2^ = 0.107, F = 4.173, *p* < 0.001). In Model 2, after adding clinical factors, the impact of being enrolled in a master’s nursing program remained significant (B = 11.959, β = 0.280, *p* < 0.001), while no clinical characteristic was significantly related to job satisfaction. This model explained 7.7% of the variance (R^2^ = 0.169, adjusted R^2^ = 0.077, F = 1.834, *p* = 0.018). In Model 3, EOL care stress, attitudes toward EOL care, and EOL care performance were added. Attitudes toward EOL care (B = 0.628, β = 0.277, *p* < 0.001) and EOL care performance (B = 0.557, β = 0.339, *p* < 0.001) emerged as significant predictors, with being enrolled in a master’s nursing program remaining significant (B = 9.727, β = 0.228, *p* < 0.001). Sex was no longer significant, and EOL care stress showed no association. The final model accounted for 29.4% of the variance (R^2^ = 0.374, adjusted R^2^ = 0.294, F = 4.663, *p* < 0.001).

## 4. Discussion

This study explored the factors influencing job satisfaction among nurses providing EOL care in tertiary and general hospitals, focusing on EOL care stress, attitudes toward EOL care, and EOL care performance. The findings revealed that EOL care performance was the strongest predictor of job satisfaction, followed by attitudes toward EOL care and educational level. In contrast, EOL care stress did not exhibit a direct association with job satisfaction.

EOL care performance was a significant determinant of job satisfaction, emphasizing that nurses’ perceived competence in providing physical, psychological, and spiritual care substantially contributes to professional fulfillment and emotional stability. Consistent with the results of previous studies, higher EOL care performance was linked to greater job satisfaction and overall well-being, particularly among nurses who frequently provided holistic or spiritual care for dying patients [24,33]. To enhance end-of-life care competencies, it is essential to account for the inherent complexity of end-of-life care. A systematic review highlights the inherent complexity of end-of-life care, where nurses confront intertwined ethical dilemmas, emotionally taxing situations, and demanding clinical decision-making [34]. Evidence showed that balancing patient autonomy, symptom management, and relational care creates substantial physical and emotional demands, while navigating communication and shared decision-making adds further structural complexity to nursing work. These substantial nursing demands underscore the need to strengthen available resources—such as education, organizational support, and structured palliative care systems—to maintain nurses’ coping capacity and ensure high-quality nursing performance [14,15].

Prior research has also indicated that EOL care performance can be shaped not only by individual competencies but also by standardized clinical protocols and supportive organizational resources [15,35,36]. As the use of electronic clinical information systems expands, standardized protocols that encompass EOL care offer an efficient method for documentation, communication, and timely intervention, thereby improving nurses’ performance and satisfaction [35,36,37]. Structured educational programs—such as simulation-based EOL care training and the End-of-Life Nursing Education Consortium (ELNEC) curriculum—can further enhance nurses’ communication, symptom management, and emotional preparedness, improving self-efficacy and reducing fear related to caring for dying patients [18,24,25]. Integrating such structured programs into routine staff development and onboarding processes may strengthen nurses’ readiness for EOL care and promote sustained quality improvements in care quality. Furthermore, Korea has long lacked clear guidelines and standardized protocols for EOL care, contributing to challenges for nurses caring for dying patients. Recently, however, efforts have emerged to develop more systematic protocols [25,36,37], underscoring the need for comprehensive, multidisciplinary educational strategies that also strengthen nurses’ spiritual care competencies [25].

Although there is a lack of research directly examining the relationship between attitudes toward EOL care and job satisfaction, growing evidence supports their close interconnection. Positive attitudes—grounded in the acceptance of death as a natural part of life—enable nurses to deliver compassionate and comprehensive care even under emotionally demanding conditions [18,20,22]. Such acceptance promotes empathy, professional growth, and organizational commitment [16,23]; in comparison, negative or avoidant attitudes—often reinforced by cultural discomfort with death or insufficient training—can increase moral distress and diminish job satisfaction [6,24]. Previous studies have also shown that greater knowledge and self-competence are associated with more favorable EOL care attitudes [11], and nurses who receive structured palliative care training demonstrate higher attitude scores, improved clinical performance, and increased job satisfaction [24,25,38]. In line with these findings, nursing managers should prioritize comprehensive EOL care education programs, reflective practice rounds, peer-support systems, and institutional policies that embed EOL care resources into everyday clinical practice. Such initiatives may enhance nurses’ attitudes and performance in EOL care while promoting sustained job satisfaction and retention.

In this study, EOL care stress was not found to be directly associated with job satisfaction, a finding that contrasts with the results of previous studies linking high stress levels to burnout and turnover intention [10,20]. Several explanations may clarify this discrepancy. Moderate stress may be perceived as an unavoidable aspect of professional caregiving, acting as a stimulus for adaptive coping and resilience rather than diminishing satisfaction [9,39]. Nurses who possess a strong sense of professional calling or well-developed coping strategies may also buffer the negative impact of stress, sustaining engagement and fulfillment in their clinical roles [2,19,37]. At the same time, EOL care often evokes both positive growth-oriented emotions and taxing moral or existential distress, suggesting that nurses may experience stress and meaning-making simultaneously rather than in mutually exclusive ways [2,37]. Even under intense stress, repeated encounters with death have been shown to help nurses reevaluate life’s meaning, contributing to personal and professional growth and reinforcing their identity as compassionate and resilient practitioners [2,9,37]. In addition, institutional resources—such as teamwork, peer support, and access to palliative care education—can ease the emotional demands of EOL care, thereby reducing stress and preserving job satisfaction [2,3,9,14,15,40].

EOL care stress remains a crucial challenge in clinical nursing, as nurses frequently encounter profound emotional strain and moral distress when caring for dying patients [2,4,16]. Unlike general occupational stress, EOL care stress is rooted in the moral and existential dimensions of caregiving and may exert indirect effects through mediating factors such as resilience, coping style, or a sense of professional calling [9,26,37,39]. The relationship between EOL care stress and job satisfaction may therefore be more complex than a simple direct association [2,14,15,20,39]. Thus, competing interpretations—viewing stress as either a source of burden or a catalyst for growth—may coexist, making the relationship between EOL care stress and job satisfaction inherently bidirectional and dynamic [2,37,39]. Future research should better analyze these pathways by differentiating between individual factors like self-efficacy, coping, and resilience, and organizational factors such as supervisor or peer support and organizational support, and include these as mediating variables [2,9,19]. Such work could provide a more comprehensive understanding of how EOL care stress interacts with personal and organizational resources to shape nurses’ professional well-being and competency. In addition, longitudinal and intervention studies are needed to examine how educational programs, resilience training, and supportive clinical environments help nurses regulate stress, strengthen coping capacity, and maintain job satisfaction while providing compassionate EOL care.

In this study, nurses who were enrolled in or had completed a master’s degree reported higher job satisfaction. Although direct evidence linking educational attainment to job satisfaction among EOL care nurses is limited, related findings help contextualize this outcome. Kondo et al. [23] found that both longer clinical experience and possession of a master’s degree were associated with more positive attitudes toward caring for dying patients, suggesting that advanced education fosters emotional maturity and professional confidence in complex care situations. Such positive attitudes have been connected to greater empathy, self-efficacy, and meaning in professional practice—key elements of job satisfaction [16,18,22]. Moreover, a strong sense of purpose and fulfillment, often cultivated through advanced study, can build resilience and help nurses remain satisfied despite the emotional challenges of EOL settings [2]. Taken together, these findings indicate that the higher job satisfaction observed among nurses with a master’s degree may reflect stronger professional identity, greater emotional competence, and self-directed motivation to deliver quality care. Future studies should further examine how advanced education influences nurses’ roles, coping strategies, and expectations in EOL care to clarify its complex relationship with job satisfaction.

To improve job satisfaction among nurses involved in EOL care, coordinated strategies at both educational and organizational levels are essential. Strengthening EOL care skills begins with structured, ongoing educational programs, including simulation-based communication training and ELNEC curricula, which develop clinical skills, emotional preparedness, and ethical decision-making. These programs foster confidence and more positive attitudes toward caring for dying patients, thereby enhancing job satisfaction. In South Korea, where dedicated EOL care education is limited in most nursing programs and many students receive minimal exposure during clinical practice [41], undergraduate curricula should more systematically integrate terminal care content—including coursework and supervised practicum experiences—to improve future nurses’ preparedness and competence in EOL care.

Equally important is the creation of supportive work environments. Hospitals should promote open conversations about death, provide counseling and debriefing opportunities, and recognize EOL expertise as a core component of professional nursing practice. Support at the unit level—such as peer-support groups, reflective practice sessions, and resilience-building activities—can help nurses process emotional stress, reduce burnout, and find meaning and professional growth through EOL care. At the organizational level, implementing standardized EOL care protocols within electronic medical records can enhance care consistency, strengthen interdisciplinary communication, and lessen the cognitive and emotional burden associated with complex clinical decisions. Collectively, these strategies can bolster nurses’ clinical performance, reduce emotional strain, and ultimately contribute to higher job satisfaction.

Future research should employ longitudinal and interventional designs to clarify the causal pathways linking EOL care stress, attitudes, and performance with job satisfaction. Longitudinal studies can illuminate how these relationships evolve with clinical experience or sustained organizational support, while intervention trials—such as resilience-building programs or team-based support initiatives—may identify effective strategies for enhancing nurses’ well-being and cultivating a resilient EOL nursing workforce. This forward-looking research is essential for informing evidence-based policies that ensure high-quality EOL care and support nurses’ professional satisfaction and psychological health.

While the results of this study provide valuable insight into the factors influencing job satisfaction among nurses who deliver EOL care, several limitations must be acknowledged. First, this study used an online convenience sample, which may have introduced self-selection bias—particularly the overrepresentation of nurses with higher motivation or academic involvement—thereby limiting representativeness. Prior studies show that traits such as conscientiousness, agreeableness, and strong learning motivation enhance professional engagement, suggesting that these nurses may have been more likely to participate in the survey [42,43]. Therefore, caution is needed when generalizing the findings, and future research should adopt probability-based sampling or expand recruitment through face-to-face surveys across diverse hospitals and regions. Second, the cross-sectional design restricts causal inference regarding relationships among EOL care stress, attitudes, performance, and job satisfaction. Longitudinal research is necessary to identify mediating factors such as resilience or compassion satisfaction. Finally, contextual and organizational variables—such as workload, staffing, and institutional support—were not analyzed but may meaningfully influence job satisfaction. Future studies should incorporate these factors through multi-site, mixed-method, or multi-level designs to provide a more comprehensive understanding of EOL care nursing.

## 5. Conclusions

The findings of this study demonstrate that enhancing nurses’ EOL care performance, fostering more positive attitudes toward EOL care, and supporting higher educational attainment are key factors associated with improved job satisfaction among hospital nurses. These results underscore the need for structured educational programs, supportive organizational environments, and standardized EOL care protocols to strengthen nurses’ competence and resilience in emotionally demanding clinical settings. In the Korean context—where most deaths occur in hospitals and hierarchical cultural norms influence clinical decision-making—efforts to reinforce EOL care competencies and expand undergraduate curricular integration are particularly important. Ultimately, policies that reduce workload burdens and provide stronger welfare systems will help sustain nurses’ well-being and contribute to improving the overall quality of EOL care.

## Figures and Tables

**Table 1:** Characteristics of the Participants (*N* = 215).

Variables	*N* (%) or Mean ± SD	Job Satisfaction	
		Mean ± SD	t/F (*p*)
***Sociodemographic***			
Age (year)	31.80 ± 6.99 (median: 29.00)		
Sex			−1.785
Female	202 (94.0)	111.40 ± 16.23	(0.076)
Male	13 (6.0)	119.69 ± 16.40	
Marital status			−1.546
Single	152 (70.7)	110.74 ± 15.71	(0.124)
Married	60 (27.9)	114.60 ± 17.92	
Religious affiliation			−1.938
No	129 (60.0)	110.15 ± 16.65	(0.054)
Yes	86 (40.0)	114.52 ± 15.55	
Educational level			
Bachelor’s degree ^a^	168 (78.1)	109.38 ± 15.71	11.759
Being enrolled in a master’s nursing program ^b^	39 (18.1)	122.79 ± 15.16	(<0.001) a < b
Master’s degree ^c^	8 (3.7)	111.63 ± 14.13	
Subjective economic status			
Low	9 (4.2)	117.76 ± 18.50	1.465
Medium	140 (65.1)	110.61 ± 15.92	(0.233)
High	66 (30.7)	113.83 ± 16.78	
***Clinical factors***			
Region of hospital			0.816
Gyeonggi-do	47 (21.9)	113.62 ± 19.44	(0.415)
Seoul	168 (78.1)	111.42 ± 15.38	
Size of hospital			1.177
General hospitals	68 (31.6)	113.82 ± 17.19	(0.240)
Tertiary hospitals	147 (68.4)	111.01 ± 15.89	
Clinical career (year)	7.77 ± 6.61 (median: 5.25)		
≤3	60 (27.9)	107.52 ± 16.10	2.233
>3 to ≤5	41 (19.1)	111.93 ± 12.73	(0.085)
>5 to ≤10	53 (24.7)	114.02 ± 19.08	
>10	61 (28.4)	114.34 ± 15.60	
Type of working unit			
General ward	123 (57.2)	111.18 ± 16.15	1.736
Intensive care unit	64 (29.8)	112.30 ± 16.01	(0.161)
Cancer care unit/Hospice unit	21 (9.8)	110.43 ± 15.70	
Emergency room	7 (3.3)	125.29 ± 21.46	
Position			−1.845
Staff nurse	158 (73.5)	110.67 ± 15.57	(0.066)
Charge nurse	57 (26.5)	115.30 ± 17.98	
Participation in education for end-of-life care			0.492 (0.623)
No	89 (41.4)	112.55 ± 16.70	
Yes	126 (58.6)	111.44 ± 16.11	
Participation in end-of-life care during clinical practice			0.377 (0.770)
0–1	46 (21.4)	113.96 ± 16.89	
2–5	87 (40.5)	110.79 ± 16.03	
6–10	38 (17.7)	111.71 ± 15.14	
>10	44 (20.5)	112.10 ± 17.60	

SD = Standard deviation, Missing data were excluded from analysis, Superscript letters (^a–c^) indicate statistically significant differences among groups based on post-hoc Scheffé test.

**Table 2:** End-of-life Care Stress, Attitudes toward End-of-life Care, End-of-life Care Performance, and Job Satisfaction (n = 215).

Measure	Total Score	
	Mean ± SD	Possible Range
End-of-life care stress	154.94 ± 21.07	40–200
Attitudes toward end-of-life care	89.56 ± 7.23	30–120
End-of-life care performance	55.72 ± 9.98	22–88
Job satisfaction	111.90 ± 16.33	33–165

**Table 3:** Correlations among the Research Variables.

Title 1	r (*p*)			
	Age	End-of-Life Care Stress	Attitude Toward End-of-Life Care	End-of-Life Care Performance
End-of-life care stress	0.213 (0.002 *)			
Attitude toward end-of-life care	−0.047 (0.491)	−0.104 (0.128)		
End-of-life care performance	0.061 (0.374)	0.151 (0.027 *)	0.264 (<0.001 **)	
Job satisfaction	0.161 (0.018 *)	0.018 (0.798)	0.354 (<0.001 **)	0.408 (<0.001 **)

* *p* < 0.05, ** *p* < 0.001

**Table 4:** Factors associated with job satisfaction using hierarchical regression analysis.

Variables	Model 1					Model 2					Model 3				
	B	SE	Beta	t	*p*	B	SE	Beta	t	*p*	B	SE	Beta	t	*p*
(Constant)	102.214	6.090		16.783	<0.001	104.351	9.635		10.831	<0.001	21.811	17.345		1.257	0.210
Age	0.150	0.209	0.064	0.719	0.473	0.223	0.331	0.095	0.674	0.501	0.112	0.291	0.048	0.383	0.702
Sex: Male (reference: Female)	10.594	4.923	0.143	2.152	0.033	9.803	5.212	0.133	1.881	0.062	7.023	4.654	0.095	1.509	0.133
Marital status: Married (reference: Single)	−0.181	3.051	−0.005	−0.059	0.953	−1.187	3.420	−0.033	−0.347	0.729	0.090	2.997	0.002	0.030	0.976
Religious affiliation: Yes (reference: No)	1.670	2.339	0.050	0.714	0.476	1.525	2.430	0.046	0.627	0.531	2.221	2.128	0.066	1.044	0.298
Educational level: Being enrolled in a master’s nursing program (reference: Bachelor’s degree)	12.876	2.982	0.302	4.318	<0.001	11.959	3.187	0.280	3.753	<0.001	9.727	2.808	0.228	3.464	<0.001
Educational level: Master’s degree (reference: Bachelor’s degree)	1.625	6.232	0.018	0.261	0.795	2.107	6.523	0.023	0.323	0.747	1.310	5.732	0.014	0.229	0.819
Subjective economic status: Low (reference: Medium)	2.536	5.470	0.031	0.464	0.643	3.039	5.844	0.037	0.520	0.604	6.119	5.215	0.075	1.173	0.242
Subjective economic status: High (reference: Medium)	3.891	2.350	0.110	1.656	0.099	4.513	2.469	0.128	1.828	0.069	1.961	2.188	0.055	0.896	0.371
Region of hospital: Seoul (reference: Gyeonggi-do)						−2.625	3.129	−0.066	−0.839	0.403	−1.636	2.750	−0.041	−0.595	0.553
Size of hospital: Tertiary hospitals (reference: General hospitals)						−1.048	2.862	−0.030	−0.366	0.715	−3.248	2.519	−0.092	−1.289	0.199
Clinical career (year): >3 to ≤5 (reference: ≤3)						2.265	3.333	0.054	0.679	0.498	4.969	2.949	0.119	1.685	0.094
Clinical career (year): >5 to ≤10 (reference: ≤3)						1.554	3.863	0.041	0.402	0.688	1.302	2.398	0.034	0.383	0.702
Clinical career (year): >10 (reference: ≤3)						−0.333	5.992	−0.009	0.055	0.956	1.192	5.255	0.033	0.227	0.821
Type of working unit: Intensive care unit (reference: General Ward)						0.503	2.682	0.014	0.187	0.852	−1.514	2.482	−0.042	−0.613	0.541
Type of working unit: Cancer care unit/hospice unit (reference: General Ward)						0.870	4.002	0.016	0.217	0.828	−2.026	3.520	−0.037	−0.576	0.566
Type of working unit: Emergency room (reference: General Ward)						9.890	7.092	0.100	1.395	0.165	0.572	6.465	0.006	0.088	0.930
Position: Charge nurse (reference: Staff nurse)						1.366	2.830	0.037	0.483	0.630	1.944	2.476	0.053	0.785	0.433
Participation in education for end-of-life care: Yes (reference: No)						−1.000	2.342	−0.030	−0.427	0.670	0.944	2.074	0.028	0.455	0.649
Participation in end-of-life care during clinical practice: 2–5 (reference: 0–1)						−2.845	3.094	−0.085	−0.920	0.259	−2.752	2.752	−0.083	−1.000	0.319
Participation in end-of-life care during clinical practice: 6–10 (reference: 0–1)						−2.638	3.895	−0.060	−0.677	0.499	−1.523	3.427	−0.035	−0.444	0.657
Participation in end-of-life care during clinical practice: ≥10 (reference: 0–1)						−3.683	3.855	−0.091	−0.955	0.341	−3.947	3.416	−0.098	−1.155	0.249
End-of-life care stress											−0.007	0.051	−0.008	−0.128	0.898
Attitudes toward end-of-life care											0.628	0.151	0.277	4.159	<0.001
End-of-life care performance											0.557	0.110	0.339	5.061	<0.001
	R^2^ = 0.141, adjusted R^2^ = 0.107 F = 4.173, *p* < 0.001					R^2^ = 0.169, adjusted R^2^ = 0.077 F = 1.834, *p* = 0.018					R^2^ = 0.374, adjusted R^2^ = 0.294 F = 4.663, *p* < 0.001 Durbin–Watson = 1.557				

## Data Availability

The data presented in this study are available from the corresponding author upon request. The data are not publicly available due to privacy or ethical restrictions.

## Abbreviation

The following abbreviation is used in this manuscript:

EOL	End-of-life
